# Radioprotective Effect of Thymoquinone in X-irradiated Rats

**DOI:** 10.31557/APJCP.2021.22.9.3005

**Published:** 2021-09

**Authors:** Sanaa A. El-Benhawy, Mohamed I. Morsi, Rasha A. El-Tahan, Noura A. Matar, Hawa MG Ehmaida

**Affiliations:** 1 *Department of Radiation Sciences, Medical Research Institute, Alexandria University, Alexandria, Egypt. *; 2 *Department of Biochemistry, Medical Research Institute, Alexandria University, Alexandria, Egypt. *; 3 *Department of Histochemistry and Cell Biology, Medical Research Institute, Alexandria University, Alexandria, Egypt. *; 4 *Faculty of Medical Technology, Tobruk University, Libya. *

**Keywords:** Thymoquinone, Nigella Sativa, Ionizing Radiation, Radioprotective agent

## Abstract

**Background:**

Thymoquinone, has anti-inflammatory, anti-oxidant, and cardio protection properties. This study aimed to evaluate the radioprotective effect of thymoquinone in whole body X-irradiated rats.

**Methods:**

This study conducted on 40 male adult Wistar albino rats randomized into the following groups: Group I: Control rats did not receive thymoquinone or ionizing radiation. Group II: Whole-body irradiated rats with 6 Gy of X-ray. Group III: Rats orally intubated with thymoquinone (10 mg/kg/day) for 7 days then subjected to whole-body irradiation with 6 Gy then supplemented with thymoquinone for another 7 days. Group IV: Rats orally intubated with thymoquinone (20 mg/kg/day) for 7 days then subjected to whole-body irradiation with 6 Gy then supplemented with thymoquinone (20 mg/kg/day) for another 7 days. LDH, CK-MB, ALT, AST, MDA, TAC, Catalase activity, GPX, GSR and GSH were measured.

**Results:**

Lipid peroxidation biomarker in the blood of X-irradiated rats significantly increased and accompanied by decrease in the levels of GSH, GSR, GPX, catalase as well as TAC. Moreover, exposure to IR significantly increases cardiac and liver enzymes. However, administration of TQ to X-irradiated rats with either 10 mg/kg or 20 mg/kg have the same reform effects and significantly protects rats against adverse effects of IR.

**Conclusion:**

Exposure to X-ray leads to significant changes in cellular biochemical and morphological conditions. Administration of TQ before radiation treatment significantly decreases the adverse effects of IR. TQ can improve cardiac function, decrease myocardial enzyme levels and inhibit oxidative stress.

## Introduction

Ionizing radiation (IR) emission is a process in which an unstable nucleus emitted the excess energy to achieve stability. IR can interact with and damage DNA of living organisms. Long-term IR exposure causes adverse effects in the body, such as cancer, skin redness, lowered immunity, and serious damage to organs and tissues due to formation of reactive oxygen species (ROS) and reactive nitrogen species (RNS) (Hosoda et al., 2021; Wang et al., 2020).

Ionizing radiation is used in radiotherapy (RT), the most popular treatment for human cancers. Approximately 80% of cancer patients will need radiotherapy at some point in their treatment, whether for curative or palliative purposes. To achieve the best results, a careful balance must be struck between the total dose of RT administered and the threshold limit of the surrounding normal tissues. To attain optimum tumor control with a higher dose, normal tissues must be protected from radiation damage (Mun et al., 2018).

Radioprotectors are compounds that reduce radiation damage on healthy tissues. These substances, which are often antioxidants, must be present before or at the time of irradiation to be effective (Kuruba and Gollapalli, 2018). A large number of compounds such as antioxidants, sulfhydryl compounds, cytoprotective agents, immunomodulators, metalloelements and vitamins have been tested to determine the extent of radioprotection and mitigation against the effects of IR (Musa et al., 2020). Radioprotectors work by different mechanisms such as scavenging of free radicals; enhancing DNA repair; synchronizing of cells; modifiying redox sensitive genes; modulating growth factors and cytokines and suppressing apoptosis (Mishra et al,, 2018).

Because of their low toxicity and high tolerability, various plants and plant-derived natural substances have been shown to be effective radioprotectors (Kma, 2014). Black seed (Nigella Sativa) oil’s main bioactive component is thymoquinone (TQ). It has promising pharmacological properties in the treatment of a variety of diseases. It had potent antioxidant, anti-inflammatory, anti-cancer, and other biologically significant properties (Gupta, 2016). Almost no side effects were observed irrespective of a large dose range, indicating a broad therapeutic window (Tekbas et al., 2018). TQ has been shown to protect against neurotoxicity caused by heavy metals and radiation in many studies (Elmaci and Altinoz, 2016, Mansour et al., 2001). Furthermore, TQ has been shown to have possible cardiovascular protective effects (Xiao et al., 2018). 

The main objective of this work was to evaluate the radioprotective effect of thymoquinone in whole body X-irradiated rats. 

## Materials and Methods

This work was conducted on 40 male adult Wistar albino rats that randomized into the following groups:

Group I: 10 control rats did not receive thymoquinone or ionizing radiation (IR), only administered olive oil as a vehicle in a volume equivalent to that given to treated animals.

Group II: 10 whole-body irradiated rats with 6 Gray (Gy)of x-ray.

Group III: 10 Rats orally intubated with thymoquinone (10 mg/kg body weight/day) for 7 days then subjected to whole-body irradiation with 6 Gy of x-ray then the rats were supplemented with thymoquinone (10 mg/kg body weight/day) for another 7 days.

Group IV: 10 Rats orally intubated with thymoquinone (20 mg/kg body weight/day) for 7 days then subjected to whole-body irradiation with 6 Gy of x-ray then the rats were supplemented with thymoquinone (20 mg/kg body weight/day) for another 7 days.

*Animals*

The animals were bought from the Medical Technology Center’s animal house at Medical Research Institute, Alexandria University. They weight 200-250 g and housed 10 per cage throughout the experiment and were maintained on a standard laboratory diet and water. All procedures were performed by the Institutional Animal Care and Use Committee (IACUC)- Alexandria University (Approval Number: AU0121950813). The research also adheres to the ARRIVE guidelines and the National Research Council’s guide for the care and use of laboratory animals.

*Preparations of thymoquinone*

Thymoquinone was obtained from Sigma-Aldrich (St Louis, MO, USA). Thymoquinone stock solution in olive oil was prepared and administered to rats by oral gavage daily for 14 days to achieve the doses of thymoquinone 10 and 20 mg/kg/day. 

*Irradiation*

In a linear accelerator (PRIMUS, Mid-Energy, Toshiba Medical Systems, Tokyo, Japan), whole-body irradiation was performed with x-ray energy outputs of dose rates of 300 Mu/min at Alexandria Ayadi Al-Mostakbal Oncology Center, Egypt. Animals were subjected to whole-body X-irradiation dose of 6Gy.

*Blood Samples Collection*

At the end of the experiment, rats were sacrificed and a blood sample was collected by heart puncture. The blood sample was separated into two aliquots. The first one left to coagulate for 15min and serum was separated by centrifugation and stored for further use. The second aliquot was collected in vials containing Ethylene diamine tetra acetic acid (EDTA) to prepare erythrocyte lysate. Alanine aminotransferase (ALT), aspartate aminotransferase (AST) activities, malondialdehyde (MDA), total antioxidant capacity (TAC), Catalase activity, Glutathione peroxidase (GPX), Glutathione reductase (GSR), and reduced glutathione (GSH) assay kits were purchased from Biodiagnostic company (Cairo, Egypt). Lactate dehydrogenase (LDH) was assayed by ELISA kit according to the manufacture instructions (MyBioSource, San Diego USA) and Creatine kinase-MB (CK-MB) was assayed by Enzyme-linked immunosorbent assay (ELISA) kit according to the manufacture protocol (Abcam, UK).

*Preparation of Erythrocyte lysate*

Blood sample of 3.0 mL was obtained in EDTA-containing vials. Erythrocytes were separated by centrifugation at 400xg for 10 minutes, then washed with normal saline several times (0.9 percent NaCl solution). Erythrocytes were resuspended in 0.9 percent NaCl solution to their original volume. The resuspended RBCs were lysed (1:10) in 10 mM sodium phosphate buffer (pH 7.4) and the lysate was stored at -70^ο^C until required.

*Histopathological study*

The liver and heart of all experimental animals were excised for the histopathological study. Small pieces of the organ were fixed in 10% neutral buffered formalin for 24 h. Washed in tap water for 24 h, dehydrated in ascending sequence of ethyl alcohol, cleared in xylene, and embedded in paraffin wax. Using rotary microtome, sections of 5 microns thick were cut and stained with Mayer’s Haematoxylin and Eosin stain. That paraffin sections were brought down to distilled water, stained with Haematoxylin for 7 minutes then counterstained with Eosin for 3 minutes, dehydrated in ascending series of alcohol, cleared in xylene, and mounted in canada balsam (Bancroft JD and Gamble M, 2013).

*Statistical Analyses*

The data was fed into a computer and analyzed with IBM SPSS 20.0 (Statistical Kit for the Social Sciences) software. The mean and standard deviation were used to characterize quantitative results. The Kolmogorov-Smirnov test was used to determine if the distributions of quantitative variables were normal. The parametric test was used since the variables have a normal distribution. An independent t-test was used to compare two independent groups. The significance of the obtained results was determined at 5% level of significance.

## Results

*Effect of TQ on Oxidative Stress Biomarkers*

*TAC and MDA*

**Figure 1:**
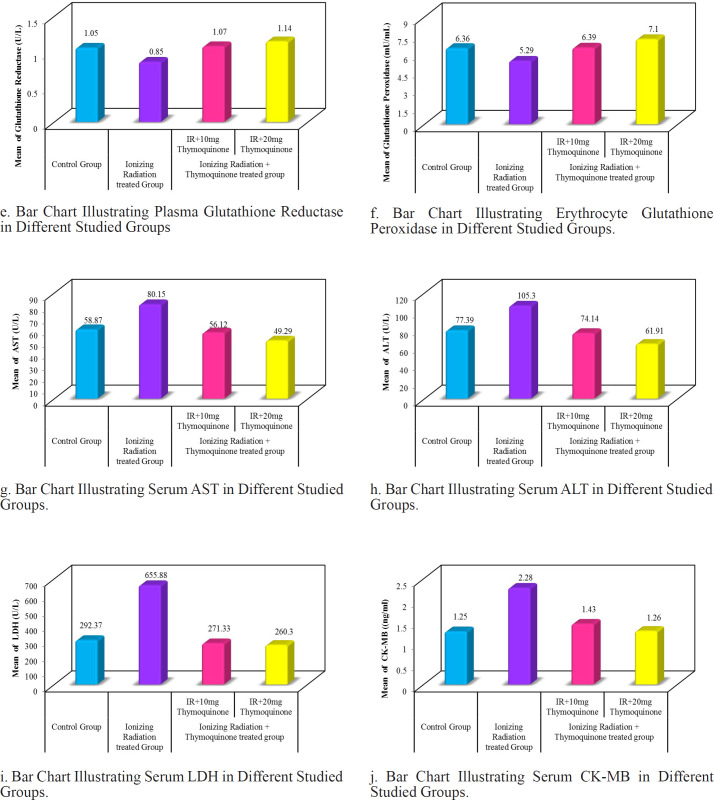
(a), Total antioxidant capacity (TAC) in different studied groups; (b), Lipid Peroxide Malondialdehyde (MDA); (c), Reduced Glutathione (GSH); (d), Plasma catalase; (e), Glutathione Reductase; (f), Erythrocyte Glutathione Peroxidase; (g), Serum AST; (h), Serum ALT; (i), Serum LDH; (j), Serum CK-MB

Range and mean±SD of plasma TAC and MDA in all studied groups are presented in Table (1) and Figure (1 a, b). TAC was significantly lower in an x-irradiated group than in the control group (p1<0.001) and significantly increased after TQ supplementation with either 10 mg/kg or 20 mg/kg (p2<0.001 and p2<0.001 respectively) and became within normal control values (p1=0.997 and 0.128 respectively). Regarding MDA, it was significantly higher in an x-irradiated group than the control group (p1<0.001) but TQ supplementation significantly decrease serum MDA levels with either 10 mg/kg or 20 mg/kg (p2<0.001 and 0.002 respectively) and became within normal control values (p1=0.935 and 0.454 respectively). 20 mg/kg TQ dose significantly improves MDA levels than 10 mg/kg (p3=0.043).

*Reduced Glutathione (GSH) and Catalase*

As presented in Table 1 and Figure (1c, d): exposure to ionizing radiation significantly depleted plasma catalase and GSH than in control group (p1=0.013 and 0.007, respectively). However, administration of TQ to x-irradiated rats with either 10 mg/kg or 20 mg/kg significantly increase plasma catalase (p2=0.011 and 0.001 respectively) and GSH (p2=0.014 and <0.001 respectively). 20 mg/kg TQ dose significantly increases plasma catalase (p3=0.039) and GSH (p3=0.031) levels than 10 mg/kg. 

*Glutathione Reductase (GSR) and Glutathione Peroxidase (GPX)*

Glutathione Reductase and Glutathione Peroxidase significantly decreased in the x-irradiated group in the control group (p1=0.049 and 0.029, respectively). However, administration of TQ to x-irradiated rats with either 10 mg/kg or 20 mg/kg significantly increases Glutathione Reductase (p2=0.018 and 0.024 respectively) and Glutathione Peroxidase (p2=0.001 and 0.015 respectively) (Table 1 and Figure (1e, f).

*Alanine aminotransferase (ALT) and Aspartate aminotransferase (AST)*

Exposure to ionizing radiation significantly increases liver enzymes AST and ALT in the control group (p1=0.002 and 0.001, respectively). However, administration of TQ to x-irradiated rats with either 10 mg/kg or 20 mg/kg significantly decreases AST (p2=0.003 and 0.001 respectively) and ALT (p2=0.009 and 0.001 respectively) serum levels (Table 1 and Figure (1g, h).

*CK-MB and LDH*

Exposure to ionizing radiation significantly increases heart enzymes CK-MB and LDH than in the control group (p1=0.001 and 0.001, respectively). However, administration of TQ to x-irradiated rats with either 10 mg/kg or 20 mg/kg significantly decreases CK-MB (p2=0.001 and 0.001 respectively) and LDH (p2=0.001 and 0.001 respectively) serum levels (Table 1 and Figure (1i, j). 

*Histopathological Results*

*Histopathology of liver*

**Figure 2:**
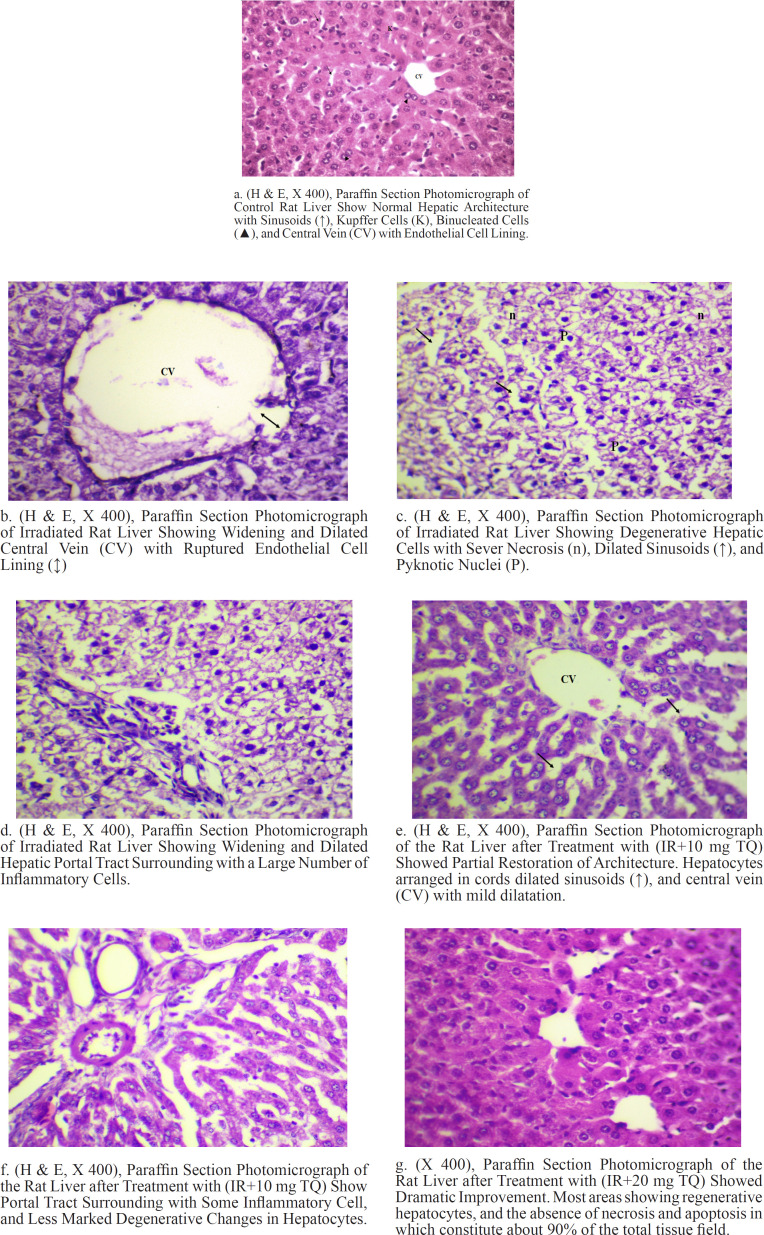
(H & E, X 400), (a), Paraffin Section Photomicrograph of Control Rat Liver Show Normal Hepatic Architecture with Sinusoids (↑), Kupffer Cells (K), Binucleated Cells (▲), and Central Vein (CV) with Endothelial Cell Lining. (b), Paraffin section photomicrograph of irradiated rat liver showing widening and dilated central vein (CV) with ruptured endothelial cell lining (↑). (c), Paraffin section photomicrograph of irradiated rat liver showing degenerative hepatic cells with sever necrosis (n), dilated sinusoids (↑), and pyknotic nuclei (P). (d), Paraffin section photomicrograph of irradiated rat liver showing widening and dilated hepatic portal tract surrounding with a large number of inflammatory cells. (e), Paraffin section photomicrograph of the rat liver after treatment with (IR+10 mg TQ) showed partial restoration of architecture. Hepatocytes arranged in cords dilated sinusoids (↑), and central vein (CV) with mild dilatation. (f), Paraffin section photomicrograph of the rat liver after treatment with (IR+10 mg TQ) show portal tract surrounding with some inflammatory cell, and less marked degenerative changes in hepatocytes. (g), Paraffin section photomicrograph of the rat liver after treatment with (IR+20 mg TQ) showed dramatic improvement. Most areas showing regenerative hepatocytes, and the absence of necrosis and apoptosis in which constitute about 90% of the total tissue field

The examination of control liver sections stained with H & E revealed that the liver showed anastomosing plates of hepatocytes radiating from the central vein. Hepatocytes were tightly packed, pink staining with round violet nuclei containing prominent nucleoli. The majority of hepatocytes contained a single nucleus and some cells were binucleated. The hepatocytes came in contact with blood sinusoids or neighboring hepatocytes and Kupffer cells were associated with sinusoidal lining cells (Figure 2a).

The examination of liver sections stained with H & E after exposure to ionizing radiation (6 Gy) revealed that the liver showed severely degenerative hepatocytes with severe necrosis, dilated sinusoids, and pyknotic nuclei. Widening and dilated central vein (CV) with ruptured endothelial cell lining (↑), and dilated hepatic portal tract surrounding with a large number of inflammatory cells were also observed (Figure 2b, c, and d).

The examination of liver sections stained with H & E after treatment with IR and10 mg TQ revealed that the liver showed partial restoration of architecture. Hepatocytes arranged in cords dilated sinusoids (↑), and central vein (CV) with mild dilatation. Portal tract surrounding with some inflammatory cell was also observed (Figure 2e, f).

The examination of liver sections stained with H & E after treatment with IR and 20 mg TQ revealed that the liver showed an obvious improvement in the liver histological structures. Wide area of the normal appearance of hepatocytes, well-developed Kupfer cell, and central vein. As well as, there is an absence of necrosis and apoptosis in which constitute about 90% of the total tissue field (Figure 2g).

*Histopathology of Heart*

**Figure 3:**
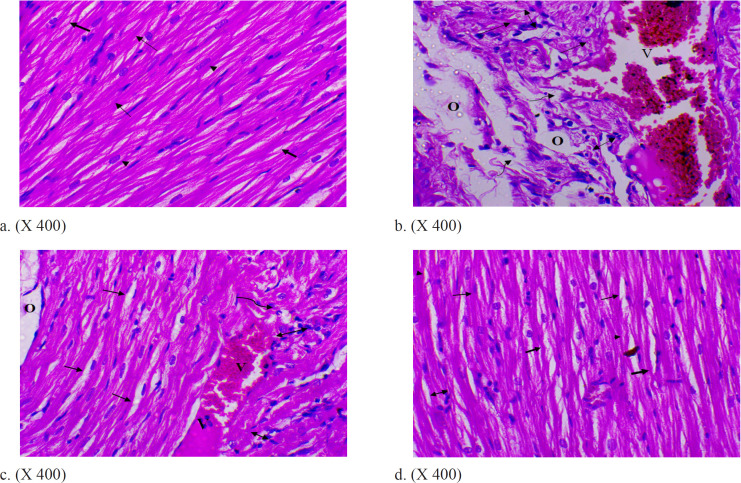
(a) (H & E, X 400), Paraffin Section Photomicrograph of Control Rat Heart Showing Branching and Anastomosing longitudinal muscle fibers (thin arrows) with central oval nuclei (arrowheads). Notice the connective tissue (thick arrow) in between muscle fibers. (b) (H & E, X 400): Paraffin section photomicrograph of irradiated rat heart showing irregular longitudinal cardiac muscle fibers (thin arrows) with areas of destructive and necrotic myocytes (wavy arrows), and interstitial mononuclear cellular infiltration (↕). Dilated congested blood vessel (V), and dilated spaces between the cardiac muscle fibers (O) are observed. (c) (H & E, X 400): Paraffin section photomicrograph of rat heart after treatment with (IRand10 mg TQ) treated showing closely adjacent and regular longitudinal cardiac muscle fibers (thin arrows) with a focal area of destructive and necrotic myocytes (wavy arrows), and dilated spaces between the cardiac muscle fibers (O). Notice the focal area of mild intercellular mononuclear cellular infiltration (↕), and less dilated congested blood vessel (V). (d) (H & E, X 400): Paraffin section photomicrograph of rat heart after treatment with (IR, and 20 mg TQ) treated rat heart showing regular longitudinal cardiac muscle fibers with prominent cross striations (thin arrows), and central oval nuclei (arrowheads). A slight mononuclear cellular infiltration (↕), and connective tissue (thick arrow) in between muscle fibers are observed

The examination of control liver sections stained with H & E showing branching and anastomosing longitudinal muscle fibers with central oval nuclei and the connective tissue in between muscle fibers (Figure 3a).

The examination of heart sections after exposure to ionizing radiation revealed that (6 Gy)-treated rat heart showing irregular longitudinal cardiac muscle fibers with areas of destructive and necrotic myocytes, and interstitial mononuclear cellular infiltration. Dilated congested blood vessels and dilated spaces between the cardiac muscle fibers are observed. (Figure 3b).

Paraffin section of (IR+10 mg TQ) treated rat heart showing closely adjacent and regular longitudinal cardiac muscle fibers with a focal area of destructive and necrotic myocytes and dilated spaces between the cardiac muscle fibers. Notice focal area of mild intercellular mononuclear cellular infiltration and less dilated congested blood vessel. (Figure 3c).

Paraffin section photomicrograph of (IR+20 mg TQ) treated rat heart showing regular longitudinal cardiac muscle fibers with prominent cross striations, and central oval nuclei. Slight mononuclear cellular infiltration and connective tissue (thick arrow) in between muscle fibers are observed. (Figure 3d).

**Table 1:** Statistical Analysis of Studied Biomarkers in Different Groups

Parameter	Control Group	IR treated Group	IR+TQ treated Group	
	(n = 10)	(n = 10)	IR+10mg TQ (n = 10)	IR+20mg TQ (n = 10)
TAC (mM/l)				
Range	1.84 – 2.25	1.13 – 1.53	1.57 – 2.01	1.81 – 2.28
Mean ± SD	2.05 ± 0.18	1.29 ± 0.15	1.83±0.15	2.01 ± 0.17
P1		<0.001*	0.997	0.128
P2			<0.001*	<0.001*
P3			0.793	
MDA (nmol/ml)				
Range	1.17 – 1.67	2.33 – 4.83	1.15 – 2.50	0.22 – 1.83
Mean ± SD.	1.34 ± 0.18	3.71 ± 0.91	1.60 ± 0.50	1.20 ± 0.48
P1		<0.001*	0.935	0.454
P2			<0.001*	0.002*
P3			0.043*	
GSH (mg/dl)				
Range	1.33 – 2.80	0.40 – 1.27	1.20 – 3.53	1.33 – 5.40
Mean ± SD.	2.01 ± 0.58	0.80 ± 0.32	2.11 ± 0.73	2.60 ± 1.27
P1		0.013*	1	0.739
P2			0.014*	<0.001*
P3			0.031*	
Catalase (U/L)				
Range	163.4–909.2	49.02–303.9	205.9–922.4	215.7–984.8
Mean ± SD.	469.2±232.1	156.8±66.03	454.3±384.9	517.6±226.0
P1		0.007*	0.746	0.349
P2			0.011*	0.001*
P3			0.039*	
Glutathione Peroxidase (mU/ml)				
Range	5.91–6.91	4.32–5.98	5.03–9.04	4.94–9.96
Mean ± SD.	6.36±0.37	5.29±0.53	6.39±0.32	7.10±2.48
				
P1		0.029*	0.179	1
P2			0.001*	0.015*
P3			0.027*	
Glutathione Reductase (U/l)				
Range	0.93–1.25	0.92–0.96	0.92–1.25	0.81–1.49
Mean ± SD.	1.05±0.12	0.85±0.01	1.07±0.11	1.14±0.21
P1		0.049*	0.696	0.998
P2			0.018*	0.024*
P3			0.513	
AST (U/l)				
Range	37.08–93.23	30.92–124.0	22.46–130.2	8.62–97.85
Mean ± SD.	58.87±24.23	80.15±33.48	56.12±37.87	49.29±31.02
P1		0.002*	0.872	0.617
P2			0.003*	0.001*
P3			0.683	
ALT (U/l)				
Range	43.91–145.8	42.09–233.9	41.18–174.8	33.91–98.45
Mean ± SD.	77.39±46.10	105.3±61.89	74.14±44.55	61.91±35.38
P1		0.001*	0.974	0.395
P2			0.009*	0.001*
P3			0.375	
LDH (U/l)				
Range	254–330	391–1039	222–303	193–291
Mean ± SD.	292.37±22.85	655.88±215.91	271.33±65.37	260.3±58.86
P1		0.001*	0.719	0.521
P2			0.001*	0.001*
P3			0.391	
CK-MB (ng/ml)				
Range	1.12–1.55	1.96–2.59	1.24–1.67	0.96–1.61
Mean ± SD.	1.25±0.16	2.28±0.23	1.43±0.19	1.26±0.40
P1		0.001*	0.491	0.813
P2			0.001*	0.001*
P3			0.51	

p1, p value for comparing between control group and other studied groups; p2, p-value for comparing between ionizing radiation treated group with Ionizing Radiation + Thymoquinone treated groups (IR+10mg and IR+20mg); p3, p-value for comparing between IR+10mg and IR+20mg; *, Statistically significant at p ≤ 0.05.

## Discussion

Cancer radiotherapy lowers the risk of recurrence and death from cancer. However, it usually includes some heart radiation exposure, and randomized studies have shown that it can raise the risk of heart disease (Taylor CW and Kirby AM, 2015). Therapeutic benefit is limited by the radiosensitivity of normal tissues adjacent to the tumor (Palumbo et al, 2019). Normal tissues respond to therapeutic radiation in a variety of ways, from mild pain to life-threatening situations. The rate at which a response occurs varies greatly from one tissue to another, and is often dependent on the dose of radiation received by the tissue. In irradiated tissue, IR is known to produce reactive oxygen species (ROS). Since water makes up 80% of the human body weight, the majority of radiation damage is caused by aqueous free radicals, which are created when radiation interacts with water. The quest for cytoprotective agents has resulted from efforts to reduce the toxicity of irradiation of normal tissue, organs, and cells. Unfortunately, the majority of radioprotectors are toxic, which limit their role in medical treatment (Cikman et al., 2014, Cikman et al., 2015).

Thymoquinone, the key component of Nigella Sativa seeds’ volatile oil, has been shown to have good antioxidant properties against oxidative damage caused by a range of free radical producing agents. Thymoquinone has been shown to have a variety of pharmacological properties, including antioxidant, hepatoprotective, neuroprotective, antidiabetic, anti-inflammatory, nephroprotective, antimutagenic, anticarcinogenic, and anticonvulsant properties in pharmacological and toxicological studies (Taysi et al., 2015, Akyuz et al., 2017, Gomathinayagam et al., 2020).

According to the findings of the current study, administration of thymoquinone (with either 10 mg/kg or 20mg/kg) pre and post-irradiation, protected the rats from adverse effects of ionizing radiation reflected by increasing TAC and decreasing MDA levels and brought their levels closer to the control group. These findings are consistent with previous reports (Safhi, 2016, Ismail et al., 2010) showed that TAC levels significantly increased with the administration of Nigella sativa (NS) intraperitoneally, which could related to the antioxidant and free-radical scavenging effects of NS. These antioxidant effects are considered to protect the tissues against radiation injury. TQ has been demonstrated to be associated with reduced ROS generation and MDA accumulation (Jrah-Harzallah et al., 2013, Lang et al., 2013). 

In the present study, exposure to ionizing radiation significantly depleted GSH levels and antioxidant enzyme systems such as plasma catalase, GPX, and GSR. However, administration of TQ to x-irradiated rats with either 10 mg/kg or 20 mg/kg significantly increases GSH and antioxidant enzyme levels. The removal of excess free radicals and hydroperoxides from the cell is typically achieved by antioxidant enzymes. Their lower levels in the irradiated group could be due to radiation-induced free radical production, which can impair the antioxidant defense mechanism, resulting in increased membrane lipid peroxidation. Due to its potent free radical scavenging action against superoxide anions and increased transcription gene responsible for the production of natural antioxidants such as catalase and glutathione peroxidase, TQ has been found to induce potent antioxidant activity. Besides, TQ can scavenge free radicals, it also can sustain the activity of specific antioxidant enzymes such as CAT, GPx, and GR (Woo et al., 2012, Gomathinayagam et al., 2020). Ahmad and Beg (2013) found that oral supplementation of TQ protects the rat liver against GSH depletion and the reduction of antioxidant enzyme activity of GPX, CAT, and GSR caused by the atherogenic suspension. Similarly, Safhi (2016) reported that after treatment with chlorpromazine, oral administration of TQ decreased lipid peroxidation and increased antioxidant enzyme levels in the rat brain, including glutathione, GPX, GSR, and CAT.

In the present study elevated levels of AST and ALT in irradiated rats were observed. Such increase may be interpreted as a result of hepatocellular damage by exposure to IR. TQ administration at all doses restored the elevated liver enzymes to normal range, indicating the protected effect of TQ against tumor cell-induced hepatotoxicity. These results agree with other studies that showed after treatment with TQ, there was a substantial recovery of hepatic damage, as evidenced by decreased plasma levels of hepatic enzymes and restored hepatic architecture. These results representing the hepatoprotective activity of TQ. These results confirmed with Mabrouk et al., (2016), a study that showed TQ reduced serum ALT and AST because of its hepatoprotective properties as thymoquinone stimulate the antioxidant scavenging enzymes system (i.e. catalase, glutathione). TQ prevented the loss of intracellular reduced glutathione and maintained the integrity of the membrane by preventing further damage to the cell membrane, as evidenced by the AST and ALT leakage. This effect is supported by histopathological changes caused by TQ as mild to moderate degree of hepatic vacuolation (Khader and Eckl, 2014; Mansour et al., 2001).

Regarding the cardioprotective effect of TQ, the present study showed that exposure to ionizing radiation significantly increases heart enzymes CK-MB and LDH than in the control group. However, administration of TQ to X-irradiated rats with either 10 mg/kg or 20 mg/kg significantly decreases CK-MB and LDH serum levels. These results are further confirmed by histopathological examination of heart tissues. Our study suggests that TQ can protect the heart against IR injury. This effect of TQ may be related to its anti-oxidative activity. In line with our result, Ahmed et al., (2013) found that, Nigella sativa oil (NSO) administration resulted in substantial normalization of physiological parameters, restoration of histological structure, and reduction of COX-2 expression in the heart. He discovered that NSO consumption can protect the heart by lowering proinflammatory cytokines, oxidative stress, and cardiac tissue damage in Pb-induced cardiotoxicity. Moreover, Gonca and Kurt (2015) found that TQ treatment reduced the infarct size. Pretreatment with TQ reduced arrhythmia scores, as well as the occurrence of ventricular tachycardia and ventricular fibrillation. TQ appears to protect against myocardial ischemia/reperfusion damage and suppresses reperfusion-induced arrhythmias.

TQ had antioxidant activity and reduced oxidative stress induced by IR, as shown by the complex thiol/disulfide homeostasis, according to Deniz et al., (2019) study. As a result, using TQ prior to radiation therapy protected the rats from oxidant side effects.

Xiao et al., (2018) demonstrated that TQ significantly improved cardiac efficiency, decreased infarct size, lowered LDH and CK-MB levels, and eliminated oxidative stress and apoptosis. Furthermore, TQ therapy promoted autophagy, which was partially inhibited by chloroquine (CQ), an autophagy inhibitor.

In conclusion, whole-body exposure to X-ray leads to significant changes in the cellular biochemical and morphological conditions, in particular, the criteria included in this study. Administration of TQ before radiation treatment at either concentration 10 mg or 20 mg have the same reform effects and significantly reduce the adverse effects of IR. TQ can effectively boost cardiac function, lower myocardial enzyme levels, and reduce oxidative stress. Confirming TQ’s cardioprotective function against IR in vivo and exploring a novel therapeutic approach for people suffering from IR injury during radiotherapy should be the subject of future research.

*List of abbreviations*

TQ, Thymoquinone; IR, Ionizing Radiation; Gy, The gray (symbol: Gy) Unit of Absorbed dose of ionizing radiation; NS, Nigella sativa; LDH, Lactate dehydrogenase; CK-MB, Creatine kinase-MB; ALT, alanine aminotransferase; AST, aspartate aminotransferase; MDA, malondialdehyde; TAC, total antioxidant capacity; GSH, reduced Glutathione; GPX, Glutathione peroxidase; GSR, Glutathione reductase; RT, Radiotherapy; IACUC, Institutional Animal Care and Use Committee. 

## Author Contribution Statement

Sanaa A. El-Benhawy (SE) research proposal idea, doing practical part of the study and the major contributor in writing the manuscript. Mohamed I. Morsi (MM) interpreting the study results and participation in manuscript writing. Rasha A. El-Tahan (RE) participation in the practical work and results interpretation and manuscript writing. Noura A. Matar (NM) performed the histological examination of the liver and heart tissues. Hawa MG Ehmaida (HE) participation in the practical work and results interpretation. All authors read and approved the final manuscript.”

## Declarations

Ethics approval and consent to participate: All procedures were performed by the Institutional Animal Care and Use Committee (IACUC)- Alexandria University (Approval Number: AU0121950813). The study also follows ARRIVE guidelines and comply with the National Research Council’s guide for the care and use of laboratory animals.

## Consent for publication

CTA sheet avaliable.

## Availability of data and material

The datasets generated and/or analyzed during the current study are not publicly available but are available from the corresponding author on reasonable request.

## Competing interests

The authors declare that they have no competing interests.
